# Combined Use of GPS and Accelerometry Reveals Fine Scale Three-Dimensional Foraging Behaviour in the Short-Tailed Shearwater

**DOI:** 10.1371/journal.pone.0139351

**Published:** 2015-10-06

**Authors:** Maud Berlincourt, Lauren P. Angel, John P. Y. Arnould

**Affiliations:** School of Life and Environmental Sciences, Deakin University, Burwood, Victoria, Australia; Institute of Ecology, GERMANY

## Abstract

Determining the foraging behaviour of free-ranging marine animals is fundamental for assessing their habitat use and how they may respond to changes in the environment. However, despite recent advances in bio-logging technology, collecting information on both at-sea movement patterns and activity budgets still remains difficult in small pelagic seabird species due to the constraints of instrument size. The short-tailed shearwater, the most abundant seabird species in Australia (*ca* 23 million individuals), is a highly pelagic procellariiform. Despite its ecological importance to the region, almost nothing is known about its at-sea behaviour, in particular, its foraging activity. Using a combination of GPS and tri-axial accelerometer data-loggers, the fine scale three-dimensional foraging behaviour of 10 breeding individuals from two colonies was investigated. Five at-sea behaviours were identified: (1) resting on water, (2) flapping flight, (3) gliding flight, (4) foraging (i.e., surface foraging and diving events), and (5) taking-off. There were substantial intra- and inter- individual variations in activity patterns, with individuals spending on average 45.8% (range: 17.1–70.0%) of time at sea resting on water and 18.2% (range: 2.3–49.6%) foraging. Individuals made 76.4 ± 65.3 dives (range: 8–237) per foraging trip (mean duration 9.0 ± 1.9 s), with dives also recorded during night-time. With the continued miniaturisation of recording devices, the use of combined data-loggers could provide us with further insights into the foraging behaviour of small procellariiforms, helping to better understand interactions with their prey.

## Introduction

Understanding and assessing the foraging behaviour of free-ranging marine animals is fundamental for determining their habitat use and responses to environmental change. However, for pelagic predators such as seabirds, it remains a constant challenge as they spend most of their time at sea, are highly mobile, and cover great distances. While at-sea distribution, species range expansion or changes in species abundance have been extensively investigated through vessel-based surveys [1–5], this method does not provide information about observed individuals’ breeding status or details of their at-sea behaviour necessary for long-term monitoring or to better understand individual species and their ecology.

Since the early 1990s, the study of the foraging behaviour and movement patterns of pelagic seabirds has been made possible by the development of miniaturized electronic technologies primarily based on VHF and satellite-tracking telemetry [6–9]. Information about foraging ecology, habitat utilisation or at-sea distribution related to the heterogeneity and the patchiness of prey resources has been published on a large range of seabird species [10–13]. However, despite these major advances in biotelemetry, some challenges remain. Most of these techniques have limitations as they rely on indirect measures of foraging effort [14–18]. Furthermore, it remains difficult to collect information about at-sea movement patterns and activity budgets in small pelagic seabird species due to the constraints of instrument size [19].

Until recently, equipment was unavailable to monitor the range of at-sea behaviours in small pelagic seabird species such as shearwaters. Most information available was collected through the use of GPS, geolocators, maximum depth gauges or time-depth recorders [20–26]. Studies combining the use of different devices to investigate simultaneous movement patterns and the allocation of time at sea for foraging (both at the surface and underwater) were restricted to large seabird species [27–31]. Animal-borne accelerometers have the potential to provide detailed information about behavioural modes of free-ranging animals because they allow recording behaviour directly [32]. Used in combination with GPS data-loggers, they are powerful tools to infer at-sea behaviour and activity budgets, with recent miniaturisation making them available for use on smaller species.

The short-tailed shearwater (*Puffinus tenuirostris*) is a highly pelagic small-sized procellariiform. It is the most abundant seabird species in Australia, with approximately 23 million individuals breeding annually during the austral spring/summer (September to April) [33]. A single egg is laid the last week of November and incubated for approximately 53 days (hatching occurs around the 3^rd^ week of January). Chick-rearing starts once the egg hatches; adults provision their chick until late March or early April [34]. While short-tailed shearwaters are listed as “Least Concern” by the International Union for Conservation of Nature (IUCN), populations in south-eastern Australia have recently experienced important decreases [35]. Despite numerous studies describing the foraging ecology [36–39] and the ecological importance of the short-tailed shearwater to the south-eastern Australian region [33], little is known about its at-sea activity budget, in particular, related to its foraging behaviour [40].

During the chick-rearing period, short-tailed shearwaters use a bimodal foraging strategy involving repeated alternation between short foraging trips (1–3 days) close to the breeding colony (within 30–70 km of the colony; [36]) and longer foraging trips (9–17 days) to distant waters of the Southern Ocean (> 1000 km; [41]). Short trips allow the adults to maximise chick-provisioning rates, while performing long foraging trips allows self-replenishment of body reserves consumed during delivery of food to the chick [41]. Assuming that higher energy costs occurring during short trips are due to greater foraging intensity [42], the aims of the present study were to investigate: 1) how individuals allocate their time at sea; and 2) to describe their diving activity during these nutritionally demanding periods.

## Materials and Methods

### Ethics Statement

The present study was conducted following the ethical guidelines of the Deakin University Animal Ethics Committee (Approval A61-2010) and in accordance with the regulations of Department of Sustainability and Environment (Victoria, Australia, Permit 10005531).

### Study sites and animal handling procedures

**Fig 1:**
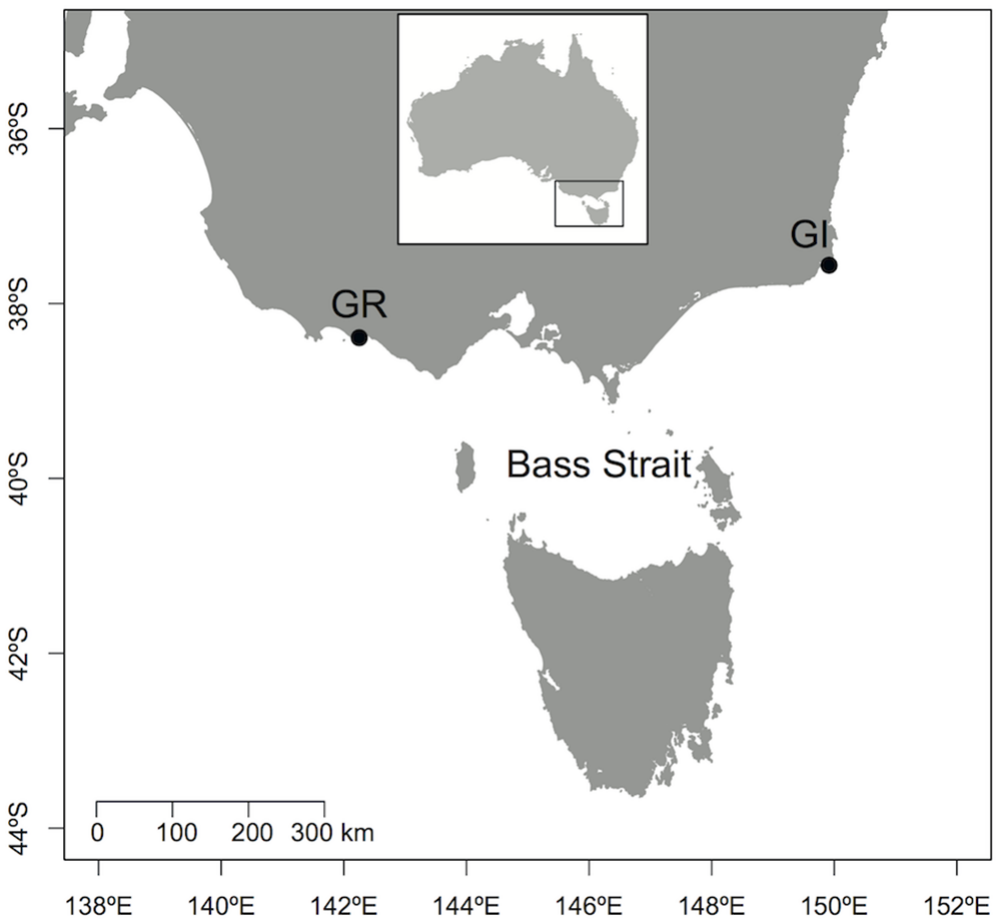
Location of Gabo Island (GI) and Griffith Island (GR) breeding colonies (closed circles) in the Bass Strait region, south-eastern Australia.

The study was conducted at Gabo Island Lighthouse Reserve (37°33’S, 149°54’E) and Griffith Island, Port Fairy (38°22’S, 142°13’E), two colonies located at both extremities of Bass Strait in south-eastern Australia (Fig 1). Breeding populations are estimated at more than 6,000 pairs at Gabo Island [43] and 15,000 pairs at Griffith Island [44]. Data were collected during the early chick-rearing period in February 2012. Burrows containing hatched chicks were monitored daily in order to identify adult attendance patterns. Chicks were weighed every night using a spring balance (± 5 g). Chicks were considered to have received a meal when their mass increased between weighing, whereas mass loss indicated no feeding event (see [41]). Both adults were considered to be performing long trips when their chicks lost weight over 5 consecutive days [37]. These burrows were targeted for GPS and accelerometer data logger deployments because the adults were likely to perform a short trip upon return. A one-way wooden trapdoor was fitted at the entrance of the burrow. Returning parents tripped a stick that closed the trapdoor when entering the burrow. Adults were left for ~30–40 minutes in the burrow to feed the chick and then captured for instrumentation.

Body mass and morphometric measurements (bill length and depth, wing length) were taken using a spring balance (± 5 g) and Vernier callipers (± 1 mm). Individuals were then instrumented with an IgotU GT–120 GPS data logger (Mobile Action Technology, Taiwan) programmed to sample location every 5 min and a tri-axial accelerometer (GCDC X6-mini, Gulf Coast Data Concepts, USA) recording acceleration at 25 Hz. A single battery was used for both loggers in order to reduce total weight and packaged in heat-shrink tubing (dimension of the devices including heat-shrink tubing: 49.0 x 24.0 x 12.0 mm). Devices were attached with black waterproof tape (Tesa AG, Germany) to feathers on the dorsal midline (total weight including the waterproof casing was 19 g, < 3.5% of the total body mass). Procedures lasted < 10 min and, after the chick was weighed, individuals were returned to the nest to resume normal behaviour. After 1 to 3 days of deployment and upon completion of at least one foraging trip, birds were recaptured in their burrows after they had fed their chick and the devices removed. In addition, a total of 50 nests at Gabo Island and 106 nests at Griffith Island were marked in study plots at the time of egg-laying in November 2011 and monitored throughout the breeding season to record the chronology of laying, hatching and fledging (and their respective success). Out of these, a total of 12 individuals at both colonies were instrumented with GPS data-loggers and accelerometers. Nest of instrumented birds were monitored after deployments and their success was compared with a *Chi*-square test to the one of the remaining nests (control group) in the studied areas. Success was not significantly different between both groups throughout the study period (Gabo Island: *χ* ^*2*^ = 0.43, *p* = 0.51 and Griffith Island: *χ* ^*2*^ = 0.39, *p* = 0.53). Consequently, the data recorded by the tri-axial accelerometers were assumed to represent normal at-sea behaviour and activity levels.

### Data processing and analysis

To investigate habitat use, GPS tracks were analysed with the *trip*, *adehabitatHR* and *adehabitatLT* packages [45, 46] within the R statistical environment [47]. An iterative forward/backward speed filter was used to remove locations yielding unrealistic high travel speeds [48] (speed threshold > 60 km·h^−1^ [37]). On average, the speed filtering removed 0.5% (range: 0–4.0%) of recorded locations during individual foraging trips. Analyses were performed on complete foraging trips, defined as the time between when individuals departed from, and when they returned to, the colony. For each foraging trip, (1) trip duration (h), (2) total distance travelled (km), (3) maximum distance to the colony (km), (4) average horizontal speed (km·h^−1^) and (5) average bearing (°) from the colony were calculated. In addition, wind speed (m·s^−1^) and wind direction (°) were used to characterize conditions experienced during foraging flight. Data were obtained from Buoyweather (http://www.buoyweather.com/index2.jsp) at two locations in the proximity of the breeding colonies (Gabo Island: 150°E– 38°S; Griffith Island: 143°E– 39°S) as 3 hourly readings and converted to daily means.

**Fig 2:**
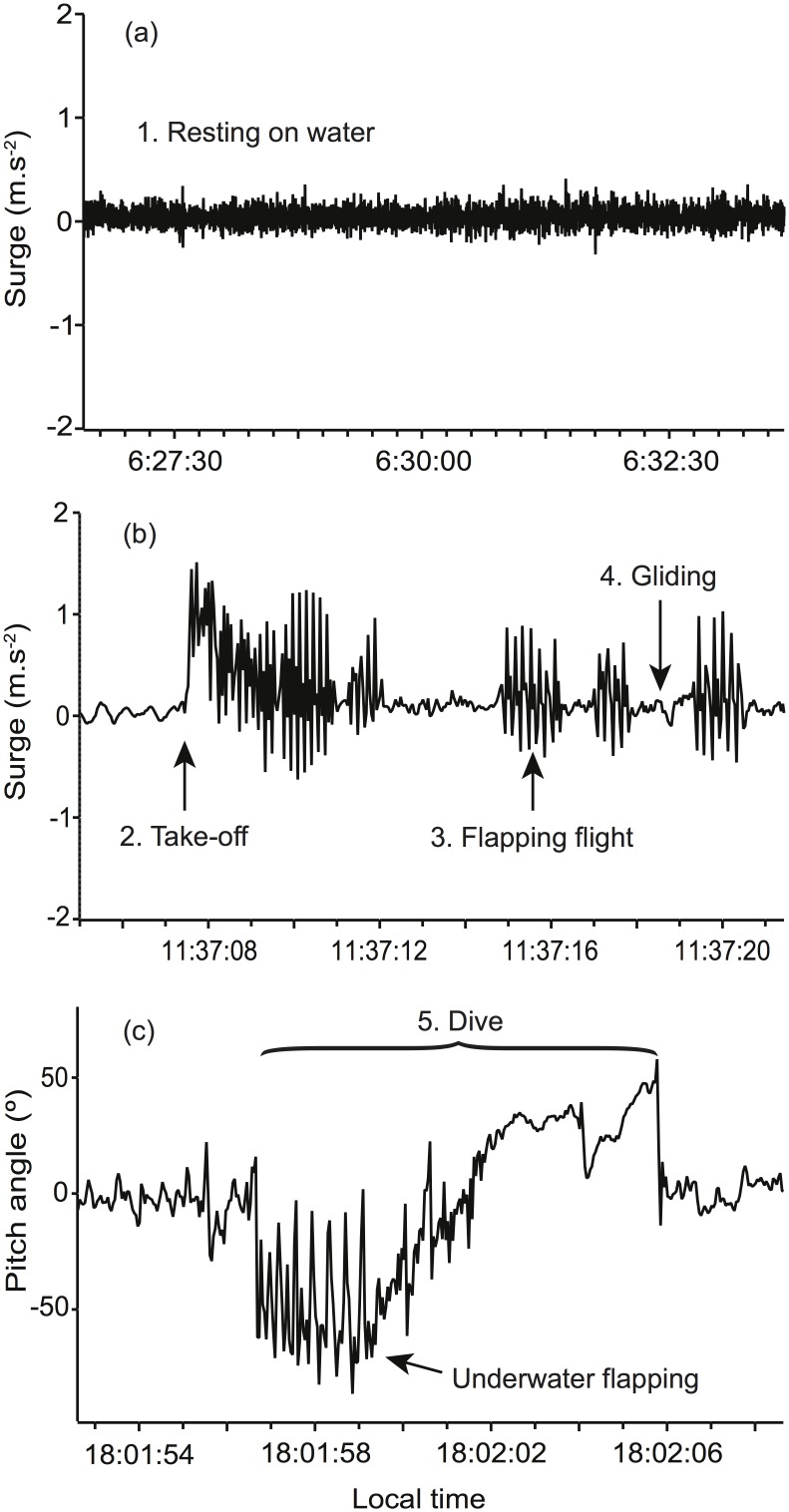
Acceleration (surge) and pitch angle profiles during the different behaviours identified. Panels represent behaviour (a) while resting at the surface of the water, (b) taking-off, flapping and gliding, and (c) while diving (underwater flapping is indicated by the arrow).

At-sea behaviours were characterized from body acceleration characteristics recorded along all three spatial axes with accelerometers measuring both dynamic acceleration (e.g. wing flapping) and static acceleration (e.g. gravity). Static acceleration was kept for behaviour determination, as the amplitude of the surge is influenced by gravitational acceleration, hence, relates to the angle of the bird [49]. Body acceleration data were analysed and classified into behaviour categories using the *Ethographer* package in Igor Pro (WaveMetrics Inc., USA). Longitudinal acceleration data (i.e. along the longitudinal axis of the body) were converted into a spectrum by continuous wavelet transformation. Each second of the spectrum was categorized into behaviour categories by unsupervised cluster analysis, using *k*-means methods [49]. Additionally, the pitch angle of the bird body was calculated from the three-acceleration signal (surge, heave and sway) using trigonometry [50]. Behaviours were classified based on previous studies of diving birds [27, 28, 51]. As the dive depth was not recorded directly by the accelerometer (i.e. no pressure sensor), individual dives were identified by a high surge along the x-axis and a negative pitch angle, following the methods of [49]. Misclassification of behaviours was then corrected based on pre-defined criteria (i.e. resting and gliding were distinguished based on speed of travel). Five activity types were identified and assumed to correspond to when the bird was taking off, in flapping flight, gliding flight, resting on the surface of the water or foraging (i.e. surface foraging or diving; Fig 2).

Trip parameters were compared between breeding colonies using non-parametric tests (Kruskal-Wallis or Wilcoxon tests) as all dependent variables differed from normal distribution. To investigate the response of at-sea behaviours to foraging trip parameters and wind conditions experienced at sea, linear mixed effects (LME) models were used with the arcsine-transformed proportions of time spent at sea (i.e. proportion of time resting or foraging) as response variables and foraging trip parameters (i.e. trip duration, total distance travelled and maximum distance to the colony) or wind parameters as explanatory variables (*nlme* package [52]). Additionally, to investigate the response of foraging trip parameters to wind conditions, LME models were used with the log-transformed foraging trip parameters as response variables and wind parameters as explanatory variables. In both cases, breeding site was held as fixed factors and bird ID was held as random factor. Only individuals performing a 1-day foraging trip were included in the models. All analyses were conducted within the R statistical environment [47]. Unless stated otherwise, data are presented as Mean ± SD and significance level was set at α = 0.05.

## Results

Some individuals could not be recaptured upon first return to the colony, either due to the friable nature of the nesting habitat preventing access to the studied burrows or because some individuals succeeded in burrowing out after they had fed the chick and avoided recapture. As a consequence, few data loggers could be recovered. Other individuals were recaptured after several short foraging trips and by this stage some data loggers were still on the birds while others had fallen off. Consequently, only 6 individuals equipped with data loggers at Gabo Island and 5 at Griffith Island were retrieved. Furthermore, due to data logger failure, data could not be downloaded for all the loggers retrieved. Complete short foraging trips were obtained from 10 individuals (see Table 1 for details) with multiple trips recorded for 2 individuals at Griffith Island (2 and 3 trips, respectively), providing a total number of 13 trips for analysis (See S1 Appendix). The average trip duration was similar for both colonies, 16.1 ± 1.3 h for individuals at Gabo Island and 19.7 ± 9.6 h for birds at Griffith Island (Wilcoxon rank sum test, W = 17, *p* = 0.62). All individuals completed a one-day trip with the exception of one bird at Griffith Island, which was away from the colony for 2 days. Although not significantly different, birds at Griffith Island travelled substantially longer distances (316.1 ± 163.3 km) than bird at Gabo Island (195.6 ± 70.5 km) (*W* = 8, *p* = 0.07). Furthermore, the maximum distance from the colony was greater for birds at Griffith Island (86.9 ± 29.9 km) compared to individuals from Gabo Island (38.8 ± 6.1 km) (W = 1, *p* = 0.002).

**Table 1:** Short foraging trip parameters in short-tailed shearwaters rearing chicks at two breeding colonies. Individuals 7 and 9 were tracked during sequential 1-day foraging trips (2 and 3, respectively) and individual 10 performed a 2-day foraging trip.

Colony	Bird ID	Body mass at deployment (g)	Trip duration (h)	Total distance travelled (km)	Maximum distance (km)	Average horizontal speed (km·h^−1^)	Average bearing (°)
Gabo Island	1	565	16.3	207.9	37.2	12.4	239.1
	2	555	15.1	118.7	38.1	6.4	24.0
	3	610	15.6	140.9	31.3	9.4	211.9
	4	565	16.5	257.9	36.3	15.3	210.9
	5	590	18.4	295.9	49.5	17.1	13.6
	6	565	15.0	152.3	40.7	9.0	21.1
	Mean ± SD	575 ± 20.7	16.1 ± 1.3	195.6 ± 70.5	38.8 ± 6.1	11.6 ± 4.1	120.1 ± 110.7
Griffith Island	7a	625	16.0	319.1	130.3	22.3	107.6
	7b	-	15.8	264.5	47.3	17.3	206.2
	8	595	17.2	306.7	68.1	19.9	267.4
	9a	565	16.0	195.7	81.1	12.4	110.5
	9b	-	15.8	181.7	71.4	11.1	118.3
	9c	-	15.4	277.9	87.1	18.7	169.3
	10	595	41.5	667.2	122.9	14.6	125.6
	Mean ± SD	595 ± 24.5	19.7 ± 9.6	316.1 ± 163.3	86.9 ± 29.9	16.6 ± 4.1	157.9 ± 60.3

**Fig 3:**
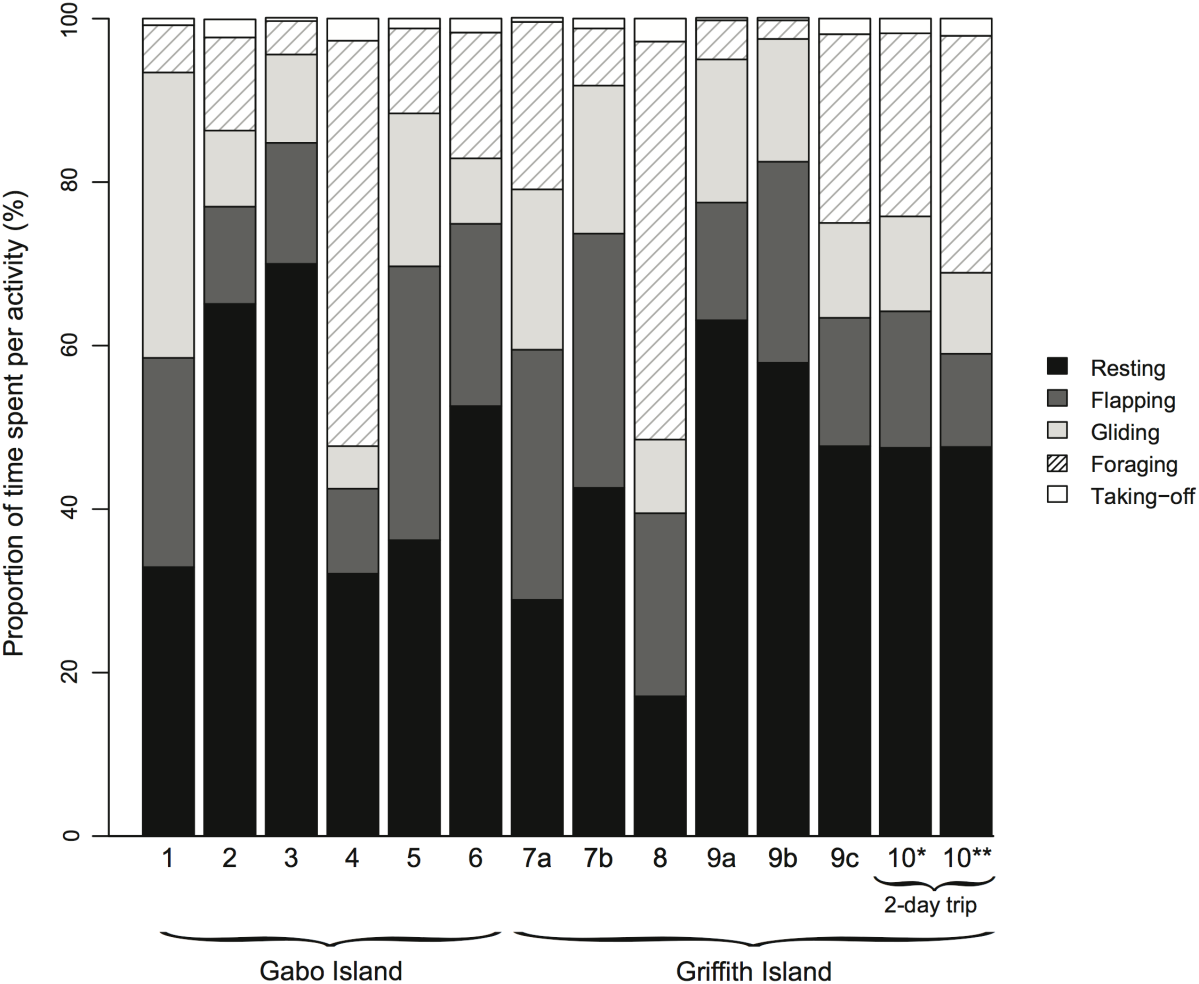
At-sea activity budget of breeding short-tailed shearwaters during short foraging trips. Proportion of time spent resting, flapping, gliding, foraging (diving and surface foraging) and taking-off during a short foraging trip. Individuals 7 and 9 were tracked during sequential 1-day foraging trips (2 and 3, respectively) and individual 10 performed a 2-day foraging trip (*activity calculated over entire trip, **activity calculated with night-time excluded).

The proportion of time spent at sea in each activity varied greatly between individuals, regardless of the breeding colony (Fig 3). Resting on water was the predominant behaviour category in all individuals (45.8 ± 15.3% of time at sea, range: 17.1–70%). Flapping flight (20.4 ± 7.8%, range: 10.4–33.5%) and foraging (including both surface foraging and diving; 18.2 ± 15.5%, range: 2.3–49.6%) contributed the second and third highest proportion of the at-sea activity budget. Intra-individual variations were also observed for the two birds tracked during sequential 1-day foraging trips at Griffith Island (Fig 3; individuals 7 and 9). For individual 7, the proportion of time spent resting on water was 28.9% and 42.6% for the respective days of tracking, and 63.1%, 57.9% and 47.7%, for individual 9, with corresponding proportion of time spent foraging of 20.5% and 7% for individual 7, and 4.8%, 2.3% and 23.1% for individual 9. For the bird (individual 10) that performed a 2-day foraging trip at Griffith Island, the proportion of time spent resting on water was similar whether night-time period was included or excluded from analyses (47.6% and 47.5%, respectively). However, the proportion of time spent in flapping flight was lower, and foraging time correspondingly higher, when the night-time period was excluded (Fig 3). This indicates that birds spent more time at night foraging than flying.

**Fig 4:**
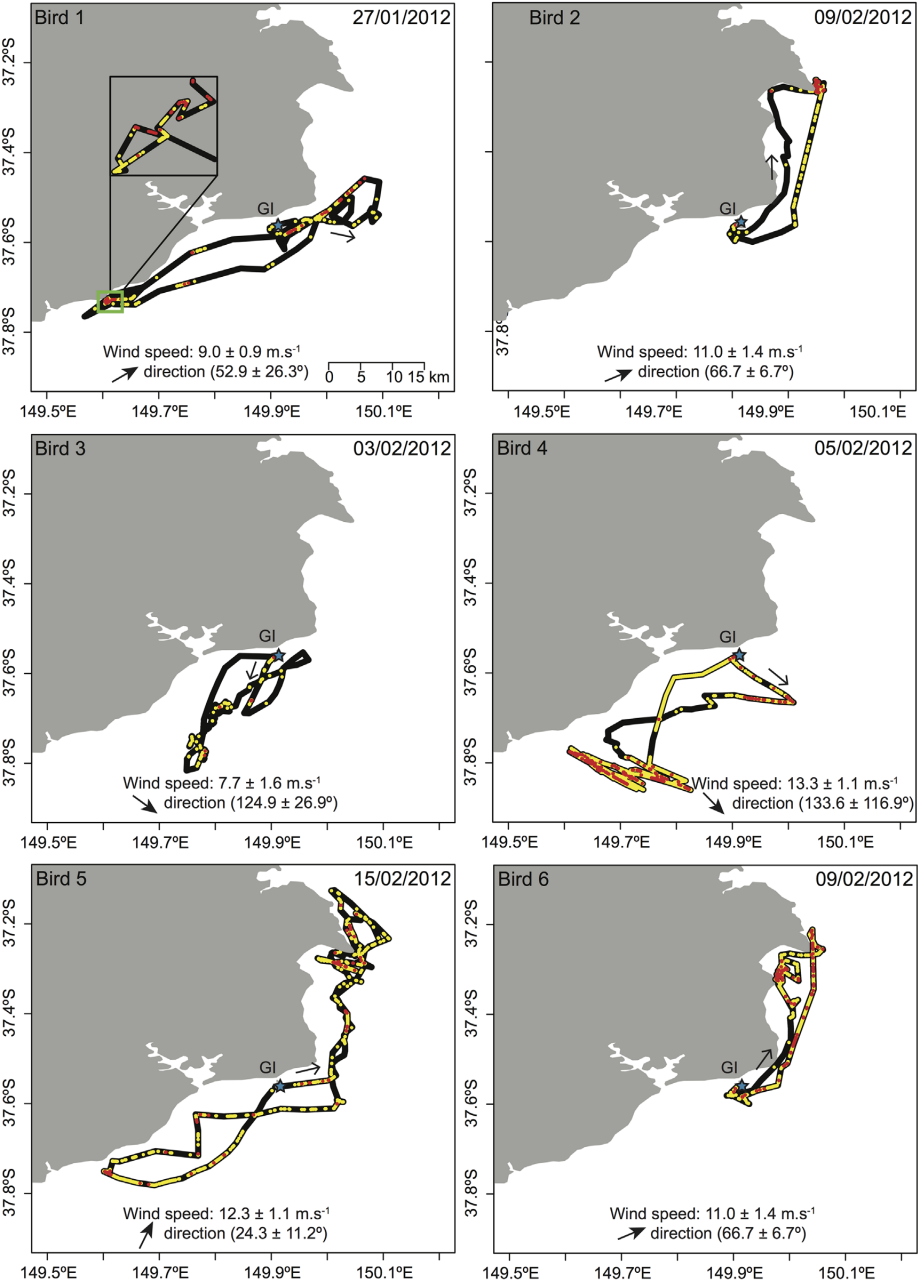
Foraging trips and related at-sea behaviour of 6 short-tailed shearwaters tracked with GPS and tri-axial accelerometers at Gabo Island. For clarity, only location of surface foraging (yellow dots) and diving events (red dots) are shown on the figure and the black line represents either resting on the water, flapping flight or gliding flight. Arrows along the tracks indicate the movement direction and the star indicates the location of the breeding colony. Wind speed and direction (mean ± SD) are indicated for each foraging trip.

**Fig 5:**
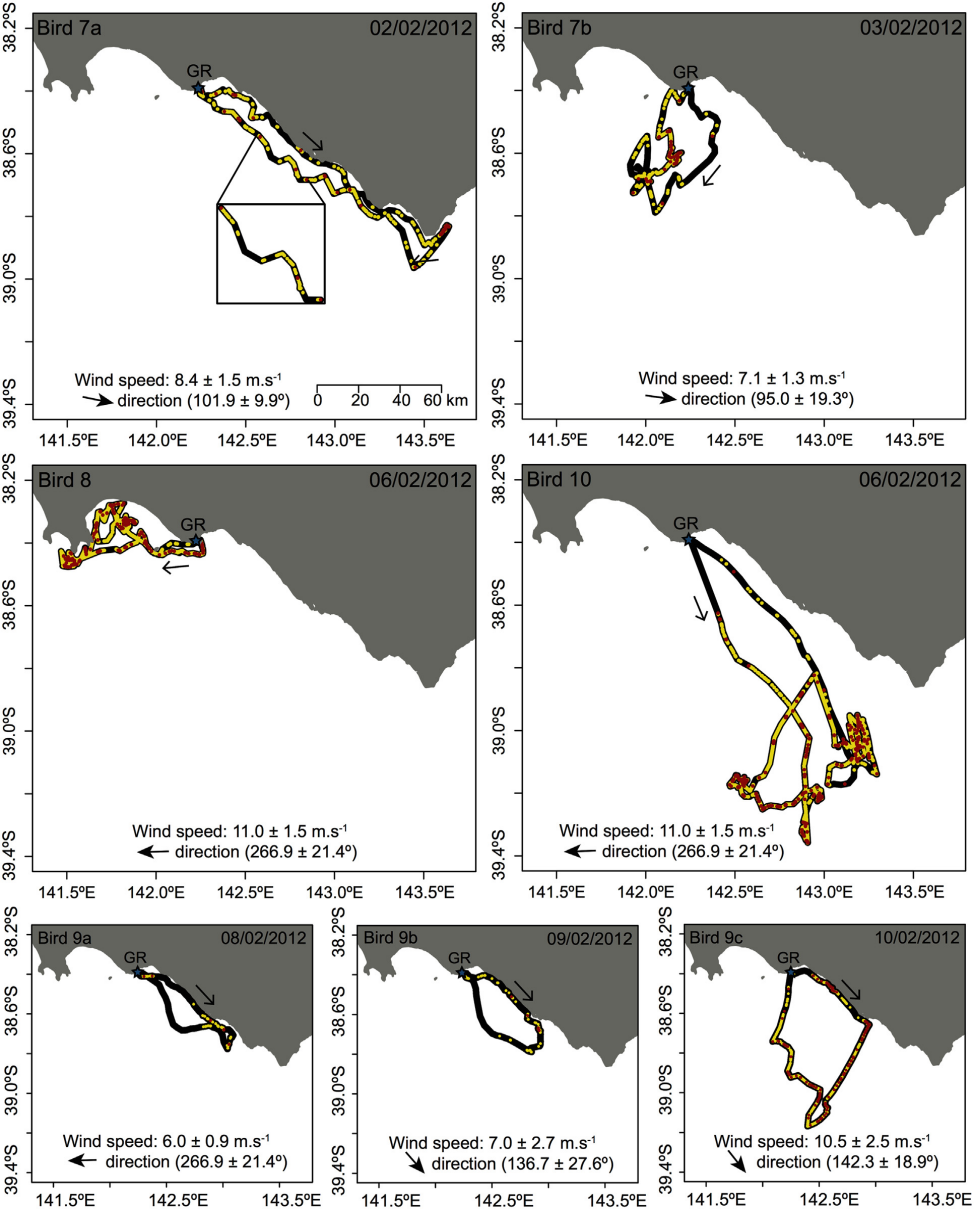
Foraging trips and related at-sea behaviour of 4 short-tailed shearwaters tracked with GPS and tri-axial accelerometers at Griffith Island. For clarity, only location of surface foraging (yellow dots) and diving events (red dots) are shown on the figure and the black line represents either resting on the water, flapping flight or gliding flight. Arrows along the tracks indicate the movement direction and the star indicates the location of the breeding colonies. Wind speed and direction (mean ± SD) are indicated for each foraging trip.

Most individuals started their outward foraging trip travelling along the coast and/or following the prevalent wind direction (Figs 4 and 5). No commuting between the breeding colonies and foraging grounds was observed with most individuals starting to surface feed or dive in areas very close to the breeding site. At Gabo Island, individuals performed their first dive on average 1.5 ± 2.3 km from the colony (range: 0.01–5.4 km) (Fig 4), 11.3 ± 9.4% of the total dives occurred within the first 10 km around the breeding colony (range: 2.7–25.0%), and 46.5 ± 36.5% of the dives events occurred further than 30 km from the colony (range: 0–93.7%). Individuals began to surface-forage within 0.2 ± 0.4 km (range: 0.002–1.0 km) of the colony and 15.1 ± 14.1% of the surface foraging events occurred within 10 km of the colony (range: 3.9–40.6%). At Griffith Island, individuals first dived within 11.7 ± 18.9 km of the breeding colony (range: 0.01–48.4 km) (Fig 5) and only 4.8 ± 4.9% of the total dives were performed within 10 km of the colony (range: 0–13.5%), while 83.1 ± 26.4% occurred further than 30 km from the colony (range: 24.4–100%). In contrast, individuals surface-foraged for the first time within 0.7 ± 1.2 km of the colony (range: 0.01 ± 2.7 km) but most of the surface foraging activity occurred further than 30 km away from the colony. Individuals were recorded to perform 76.4 ± 65.3 dives per short foraging trip (range: 8–237), with an average dive duration of 9.0 ± 1.9 s (range: 6.0–11.6 s). While individual 10, which conducted a 2-day trip, spent a considerable proportion of time (6.7%) surface foraging at night, few dives were recorded at this time.

**Fig 6:**
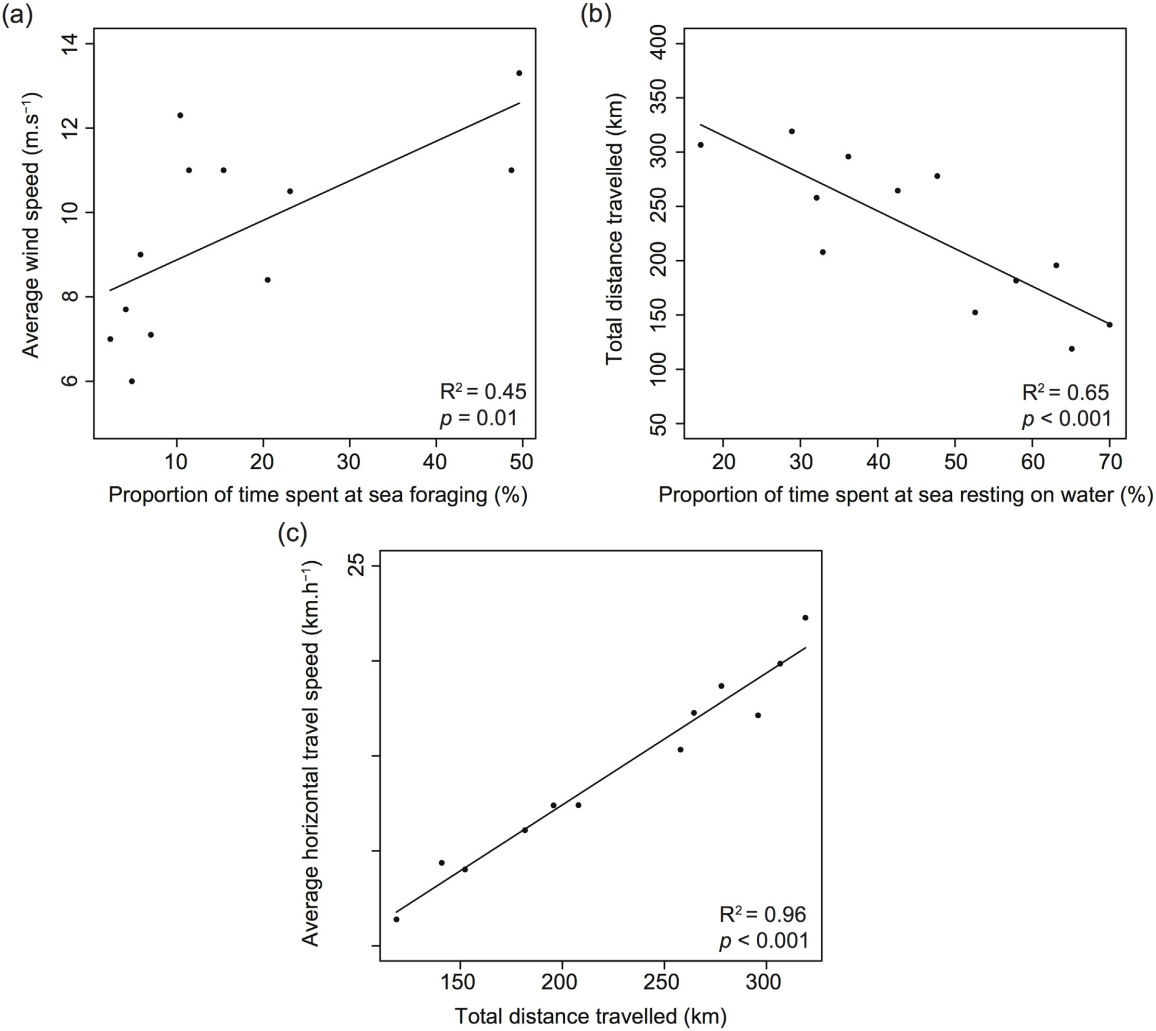
Relationships between foraging parameters, at-sea activity and wind patterns during 1-day short foraging trips in short-tailed shearwaters (n = 12). (a) Relationship between the proportion of time spent at sea foraging and the average wind speed. (b) Relationship between the proportion of time spent at sea resting on water and the total distance travelled. (c) Relationship between the total distance travelled per foraging trip and the average horizontal travel speed.

During the study period, the daily wind strength observed ranged from 21.6–47.9 km.h^−1^. The proportion of time spent foraging was positively correlated to average wind speed (*F*_1,10_ = 9.9, *p* = 0.01) (Fig 6a) while the proportion of time spent resting was negatively correlated to the total distance travelled (*F*_1,10_ = 21.7, *p* < 0.001) (Fig 6b). Finally, while the proportion of time spent in flapping flight was not correlated to horizontal travel speed (*F*_1,10_ = 2.8, *p* = 0.12), the total distance travelled per foraging trip was positively correlated to horizontal travel speed (*F*_1,10_ = 253.3, *p* < 0.001) (Fig 6c).

## Discussion

Previous studies have suggested that short-tailed shearwaters rely mainly on surface feeding and diving techniques to forage (i.e. seizing prey at the surface or pursuit diving from the surface) [40, 41, 53], as has been documented in other shearwater species using information obtained from at-sea observations [21], capillary tubes measuring maximum diving depths [20], or diving patterns recorded by time-depth data-loggers [22, 24]. However, for the first time fine-scale information on the at-sea behaviour of short-tailed shearwaters performing short foraging trips is described in the current study. The results of the present study have revealed high levels of inter-individual variation in at-sea behaviour patterns and spatial distribution of foraging activity at both colonies sampled.

While the maximum range differed between the two colonies, most likely reflecting differences in local prey distribution and weather patterns (see below), individuals from both sites displayed great variation in the proportion of time spent foraging and the number of dives made. This could reflect the high level of temporal and spatial patchiness of prey distribution for the species [54, 55]. Indeed, while differences in sampling dates could be a factor in the observed inter-individual variation in activity budgets, individuals instrumented the same day at Griffith Island (birds 8 and 10) displayed different foraging routes and very distinct at-sea activity patterns. Similarly, great differences were observed in foraging route and activity levels in one of the animals tracked for two consecutive foraging trips (bird 7). The other individual tracked for consecutive trips (bird 9) returned to the same area on each occasion, displaying different levels of activity, before exploring new areas on the third return journey to the colony. This is consistent with previous studies of short tailed shearwaters that found some individuals may, on consecutive trips, re-visit foraging areas previously exploited [36].

Surprisingly, in contrast to previous studies on short-tailed shearwaters [36] and other shearwater species [56, 57], the present study did not find individuals to have marked outward/inward commuting phases. Most individuals commenced surface foraging and/or diving shortly after they left their respective breeding ground, suggesting prey were located very close to the shore. Whereas some diving behaviour was concentrated in areas with high tortuosity and reduced travel speed in the GPS tracks, suggestive of area-restricted-search behaviour [14, 58], in all individuals diving and surface foraging were observed spread out along the entire foraging path. While it is possible that the 5 min GPS sampling interval may have precluded some areas of high tortuosity to be detected, the fact that individuals spent most of the time at sea resting on the surface (> 45%) between feeding bouts suggests that short-tailed shearwaters may search for prey patches in flight, land on the water and feed from the surface or by duck diving, possibly using the surface current to drift passively to the next profitable area. This is in contrast to observations in streaked (*Calonectris leucomelas*) and sooty shearwaters (*Puffinus griseus*), where individuals typically perform very few dives per day while spending > 75% of their time at sea flying [56, 57]. This difference may either reflect a high local abundance in prey availability near short-tailed shearwater colonies during the study or be accounted for the substantial inter-individual variations observed in at-sea activity patterns.

Individuals from both breeding colonies performed dives and surface feeding throughout the foraging trip. However, the number of dives per day was found to vary greatly between individuals. Comparable observations with similar sized shearwaters were recorded in streaked shearwaters instrumented during chick-rearing (range: 0.5–17.0 dives per day) [56] and great shearwaters (*Puffinus gravis*) instrumented during post-laying foraging trips (range: 3–156 dives per day) [24]. Furthermore, whereas previous studies of this and other shearwater species have suggested individuals mainly rest on the sea surface at night [24, 59], the observation of night-time dives (throughout the night, not only near dawn and dusk) and surface foraging activity from the single individual that conducted a 2-day trip suggests that the short-tailed shearwater is not exclusively a daytime forager. Similar observations of night diving have recently been recorded for wedge-tailed shearwaters (*Puffinus pacificus*) [60] and could reflect the availability of diel vertically migrating prey close to the surface making foraging energetically profitable at this time.

While numerous studies have documented maximum dive depths in procellariiforms [20, 22, 25, 57, 61], few were able to report dive durations [60]. Although dive depths were not obtained in this study, the short mean dive duration of 9 s recorded could indicate shallow dives near the surface. This is considerably shorter than that recently observed in the smaller (320–510 g; [60]) wedge-tailed shearwater (16–48 s) but similar to that in the larger (770–970 g) great shearwater (6–8 s, [24]). Furthermore, whereas substantial variation in dive duration was observed in both wedge-tailed and great shearwaters (SD 4–32 s), short-tailed shearwaters displayed remarkably little variation in their time submerged (SD 1.9 s). These observations (both mean duration and variability) suggest that sufficient prey were consistently available close to the surface during the present study.

The present study found that the proportion of time spent foraging at sea was positively correlated to the average local daily wind speed during the trip. This could indicate that stronger winds, likely to facilitate gliding flight (less energetically expensive and potentially faster than flapping flight), allow individuals to devote more time on the water to forage. In addition, most of the individuals used tail- or cross-winds on their outward journey, suggesting a strategy to reduce flight costs [32, 62, 63]. This highlights the important influence of wind patterns on the behaviour and foraging effort of procellariiforms [64] and is consistent with the notion that they use weather patterns to maximise their at-sea movements and minimise the cost of transport [65].

In conclusion, the results of the present study provide valuable insights into the fine scale three-dimensional at-sea behaviour of short-tailed shearwaters during their short foraging trips, periods of high parental effort. Additional studies are needed to combine these data with information on parental foraging success (i.e. adult mass gain after a foraging trip and/or chick body condition) and reproductive output in relation to environmental variability. The study further highlights the potential of simultaneous GPS and accelerometry data collection to investigate the at-sea activity budgets and, thus, energy expenditure of seabirds. This is especially important in understanding how foraging trips are organised with respect to the constant and conflicting constraints of chick-provisioning and self-feeding.
